# Factors affecting antimicrobial activity of MUC7 12-mer, a human salivary mucin-derived peptide

**DOI:** 10.1186/1476-0711-6-14

**Published:** 2007-11-11

**Authors:** Guo-Xian Wei, Alexander N Campagna, Libuse A Bobek

**Affiliations:** 1 Department of Oral Biology, School of Dental Medicine, University at Buffalo, SUNY, 3435 Main Street, Buffalo, USA, NY 14214

## Abstract

**Background:**

MUC7 12-mer (RKSYKCLHKRCR), a cationic antimicrobial peptide derived from the human low-molecular-weight salivary mucin MUC7, possesses potent antimicrobial activity *in vitro*. In order to evaluate the potential therapeutic application of the MUC7 12-mer, we examined the effects of mono- and divalent cations, EDTA, pH, and temperature on its antimicrobial activity.

**Methods:**

Minimal Inhibitory Concentrations (MICs) were determined using a liquid growth inhibition assay in 96-well microtiter plates. MUC7 12-mer was added at concentrations of 1.56–50 μM. MICs were determined at three endpoints: MIC-0, MIC-1, and MIC-2 (the lowest drug concentration showing 10%, 25% and 50% of growth, respectively). To examine the effect of salts or EDTA, a checkerboard microdilution technique was used. Fractional inhibitory concentration index (FICi) was calculated on the basis of MIC-0. The viability of microbial cells treated with MUC7 12-mer in the presence of sodium or potassium was also determined by killing assay or flow cytometry.

**Results:**

The MICs of MUC7 12-mer against organisms tested ranged from 6.25–50 μM. For* C. albicans*, antagonism (FICi 4.5) was observed for the combination of MUC7 12-mer and calcium; however, there was synergism (FICi 0.22) between MUC7 12-mer and EDTA, and the synergism was retained in the presence of calcium at its physiological concentration (1–2 mM). No antagonism but additivity or indifference (FICi 0.55–2.5) was observed for the combination of MUC7 12-mer and each K^+^, Na^+^, Mg^2+^, or Zn^2+^. MUC7 12-mer peptide (at 25 μM) also exerted killing activity in the presence of NaCl, (up to 25 mM for *C. albicans *and up to 150 mM for *E. coli*, a physiological concentration of sodium in the oral cavity and serum, respectively) and retained candidacidal activity in the presence of KCl (up to 40 mM). The peptide exhibited higher inhibitory activity against *C. albicans *at pH 7, 8, and 9 than at pH 5 and 6, and temperature up to 60°C did not affect the activity.

**Conclusion:**

MUC7 12-mer peptide is effective anticandidal agent at physiological concentrations of variety of ions in the oral cavity. These results suggest that, especially in combination with EDTA, it could potentially be applied as an alternative therapeutic agent for the treatment of human oral candidiasis.

## Background

Cationic antimicrobial peptides (CAMPs) are one of the body's host defenses against invading microorganisms. These peptides have broad-spectrum antimicrobial activity, including antibacterial, antiviral, and antifungal activity. They have a rapid onset of killing, cidal activity, and concomitant broad anti-inflammatory activities [1]. CAMPs show little or no toxicity towards mammalian cells, and have a low tendency to elicit resistance, and have thus become a promising novel group of antibiotics [2].

MUC7 peptides are the CAMPs derived from the human low molecular weight salivary mucin MUC7, a component of innate immunity (the first-line of the host defense system against pathogens). MUC7, the low molecular weight human salivary mucin glycoprotein (357 aa residues), protects the oral cavity from microbial infections through more general protective mechanisms rather than the direct killing of microorganisms. The peptides derived from the N-terminal region of MUC7 have a significant fungicidal activity *in vitro *[3-6]. The representative MUC7 peptide, MUC7 12-mer (RKSYKCLHKRCR), a cationic peptide spanning residues 40–51 of the MUC7, possesses potent antimicrobial activity in low-ionic-strength buffers and in low salt RPMI 1640 medium. This peptide exhibits synergistic antifungal effects with histatin 5 12-mer (Hsn5 12-mer) and with miconazole [7].

In effort to establish a transition from *in vitro *activity of MUC7 12-mer into clinical efficacy, it is necessary to investigate the factors affecting antimicrobial activity of MUC7 peptides. Our previous study showed that the peptides face the challenge of the degradation by proteases in saliva. We have demonstrated that antifungal activity of MUC7 12-mer-L in human whole saliva was enhanced in the presence of protease inhibitors and EDTA, and MUC7 12-mer-D isomer exerted higher antifungal activity compared to MUC7 12-mer-L in human whole saliva [8]. Beside proteases, salt sensitivity of antimicrobial peptides poses a major obstacle in their development as novel antibiotics. In addition, other factors such as pH value and temperature may also affect the antimicrobial activity of the peptides.

In this study we examined the effects of monovalent (Na^+ ^and K^+^) and divalent (Ca^2+^, Mg^2+^, and Zn^2^^+^) cations, EDTA (a chelator of divalent cations), pH, and temperature on the antimicrobial activity of the MUC7-12-mer, with a special emphasis on the activity against *C. albicans*.

## Methods

### Peptides and chemicals

MUC7 12-mer (RKSYKCLHKRCR, amino acids 40–51 of the parent human salivary mucin, MUC7) and Hsn5 12-mer (AKRHHGYKRKFH, amino acids 4–15 of the parent human salivary histatin 5) were custom-synthesized by Bio-Synthesis (Lewisville, Texas). The company analyzed the prepared peptide by high-performance liquid chromatography and mass spectrometry. The purity (>70%) was taken into consideration in preparing the stock solution of each peptide for antifungal assays. The peptide was dissolved in sterile de-mineralized water at 10 mg/mL; aliquots were freeze-dried and stored at -20°C. For each experiment, the freeze-dried peptides were re-dissolved at 1 mg/mL in sterile dd-water. The stock solutions of 1 M each EDTA, NaCl, KCl, CaCl_2_, MgCl_2_, and Zn_2 _SO_4 _were prepared in de-mineralized water. The working solutions were obtained by diluting the stock and filter-sterilization.

### Microbial strains and growth media

*C. albicans *DIS (clinical isolate from denture induced stomatitis), *Escherichia coli *HB101 and *Streptococcus mutans *UA159 were stock strains from our lab. The strains were stored at -80°C in glycerol. For each experiment, *C. albicans *cells were cultured freshly from frozen stock on Sabouraud dextrose agar (SDA, Difco) for 24 hours at 37°C. To prepare fungal cell suspension for antifungal activity assays, colonies were picked from the plate and resuspended in 10 mM sodium phosphate buffer (pH 7.4) for killing assay, and in 12.5% Sabouraud dextrose broth (SDB) for MIC assay. A 25% brain heart infusion (BHI) was used for bacterial susceptibility test (MIC test). To prepare *E. coli *cell suspension for killing assays, the overnight culture was harvested by centrifugation (3783 × g, 10 min), washed once with 10 mM sodium phosphate-buffered saline (PBS, pH 7.2) and resuspended in PBS. The concentrations of the microbial cells were adjusted to 1 × 10^5 ^cells/mL.

### MIC susceptibility tests

The effect of salt concentration on the antimicrobial activity of the peptides was tested by determining the MICs of the peptides at various cation concentrations. MICs of MUC7 12-mer were determined by the microdilution method as described previously with some modifications [7]. Briefly, two-fold serial dilutions of test agents were prepared, with 12.5% SDB at a volume of 200 μl per well, in 96-well flat-bottom microtiter plates (Costar, Cambridge, MA). The final concentration of the antifungal agents ranged from 3.13 to 50 μM for MUC7 12-mer, and 2.5 to 160 μM for cations or EDTA. The microtiter plate was inoculated with cell suspension (final concentration 1 × 10^4 ^cells per mL) and incubated at 37°C for 48 h. Afterwards, the absorbance was measured at 595 nm by using a microplate reader (Model AD 340, Beckman Coulter) to assess the cell growth. Minimal inhibitory concentrations (MIC) were determined at three endpoints: MIC-0, MIC-1 and MIC-2, indicating the lowest agent concentration showing no more than 10%, 25% and 50% growth respectively, in comparison to that of the agent-free control. For each set of conditions, the MIC tests were carried out independently two or three times, using duplicate samples each time. The MICs were reported as the geometric means and the 95% confidence intervals for the means.

The effect of pH on the antimicrobial activity of MUC7 12-mer was tested by determining the MIC of the peptide at a variety of pH values. This was achieved by altering the pH of the media with 5 M HCl or NaOH. The peptide was tested in pH conditions from pH 5 to pH 9.

The effect of temperature on the antimicrobial activity of MUC7 12-mer was tested by determining the MICs of the peptides after incubation at temperatures 60 and 90°C for 30 min followed by cooling to room temperature.

### Synergy tests

Checkerboard titrations were used to test the interaction of MUC7 12-mer peptide separately with each divalent cation, EDTA, and EDTA in the presence of divalent cations. The tests were performed in 96-well microtiter plates. A two-dimensional checkerboard (8 × 8) microdilution technique was selected to span from synergy to antagonism.

The assay were performed in the 96-well microtiter plate in total volume of 200 μl per well. In one dimension, a two-fold serial dilution of CaCl_2_, MgCl_2_, or Zn_2 _SO_4 _in sterile water was prepared across the rows, giving the final concentrations from 2.5 to 160 μM for divalent cations and EDTA, and 1–125 mM for monovalent cations. In the second dimension, a two-fold dilution series of MUC7 12-mer was prepared down the columns, giving a final concentration from 0.78 to 100 μM. Each well of the microtiter plate was inoculated with *C. albicans *DIS cells suspended in 25% SDB (2 × 12.5%) to a final cell concentration of 1 × 10^4 ^cells per mL. For bacteria, *S. mutans *or *E. coli *cells were suspended in 50% BHI (2 × 25%) to a final cell concentration of 1 × 10^5 ^cells per mL. After incubation at 37°C for 48 h, the absorbance of the culture was measured at 595 nm by using a microplate reader. MIC was determined at endpoint MIC-0 and the values were used for calculating the fractional inhibitory concentration (FIC), defined as the ratio of the MIC of a drug used in combination to the MIC of the drug tested alone.

### Synergy tests interpretation

The FIC index (FICI, the sum of the FICs) was calculated as described by Meletiadis *et al*. 2005 [9]. Briefly, for each checkerboard assay there were several ΣFICs, ranging from the ΣFIC_min _(the lowest ΣFIC) to ΣFIC_max _(the highest ΣFIC). The reported FIC index (FICi) was the ΣFIC_min _when the ΣFIC_max _was lower than 4; when ΣFIC_max _was higher than 4, the ΣFIC_max _was reported as FICi. The ΣFIC_max _was also reported as the FIC index for data sets where the ΣFIC_max _was lower than 4 but the ΣFIC_min _was higher than 1. If for a data set the ΣFIC_min _was lower than 0.5 and the ΣFIC_max _was higher than 4, both ΣFIC were reported. The off-scale MIC was converted to the next highest or lowest two fold concentration. The data represents three independent experiments. As the FICi are not normally distributed, the median and the range of FICi among the replicates were calculated. FICi values = 0.5 have been considered to indicate synergism; 0.5–1.0, additivity; 1–4, indifference; and > 4, antagonism.

### Killing assay

The assays were performed in a final volume of 40 μl. Two-fold serial dilutions of NaCl (final concentrations ranging from 6.25 to 150 mM) and MUC7 12-mer or Hsn5 12-mer (at a final concentration of 25 μM in each reaction) were prepared in 10 mM sodium phosphate buffer (PB), pH 7.4, in a volume of 20 μl. An equal volume (20 μl) of *C*. *albicans *DIS or *E. coli *HB101 suspended in the same buffer was then added (to a final concentration of 5 × 10^4 ^cells/ml). After incubation at 37°C for 1.5 hours, the samples were diluted 20-fold with the same buffer and 50 μl aliquots (approximately 120 cells) of each sample were plated on tryptic soy agar for *E. coli *and SDA for *C. albicans*. The plates were incubated at 37°C for 24 h aerobically. The number of colony-forming units (CFUs) was counted. Loss of cell viability (percentage of killing compared to agent free control) was calculated as (1- amount of viable cells in the test group)/(amount of viable cells in the control group) × 100.

### Flow cytometric assay

Flow cytometric assays were based on detection of increased permeability of fungal cells to propidium iodide (PI), a membrane impermeant DNA-intercalating dye, following treatment with MUC7 12-mer peptide in the presence of KCl. Analyses were performed on a Becton Dickinson FACScalibur system equipped with an argon-cooled argon laser (488 nm, 5 mW) and standard system configuration for orange-filtered light detection (620 nm) using CELLQuest software (Becton Dickinson, Heidelberg, Germany). For these analyses, two-fold serial dilutions of KCl ranged from 5 to 80 mM were prepared in 10 mM sodium phosphate buffer (Na-PB), pH 7.4, containing 2% glucose (PBG). MUC7 12-mer was added to each tube, giving a final concentration of 25 μM in (0.2 mL). *C. albicans *cells subcultured on SDA were suspended in PBG to give 3 × 10^6 ^cells/mL, and aliquots (0.1 mL) of the fungal suspension were then added to the solution containing the peptide and KCl. After incubation at 37°C for 1.5 h, a 1 mM-filtered solution of PI (Sigma-Aldrich) was then added to the peptide-treated fungi at a final concentration of 3 mg/L. The cell suspension without K^+ ^and peptide was used as the agent free control. The samples were analyzed after 4 min of incubation at 37°C. The cell scattergram and the intensity of fluorescence at FL3 (red fluorescence, 620 nm) were recorded by using a logarithmic scale. The results are expressed as the percentage of positive cells showing high fluorescence at FL3. To obtain a positive control for permeabilization, fungal suspensions were pelleted and resuspended in cold absolute ethanol for 30 min at -20°C. Ethanol was removed by aspiration following centrifugation at 1000 g for 10 min, and the pellet resuspended in PBG solution. Data analysis was performed with the FCS Express version 3 (De Nova Software, Thornhill, Ontario, Canada).

## Results

### Growth inhibitory activity by MUC7 12-mer in the presence of salts or EDTA

The effect of salts or EDTA on the antimicrobial activity of MUC7 12-mer was examined by determining the FICi values of each combination pair in the checkerboard. As shown in Table 1 for *C. albicans*, the MICs of MUC7 12-mer were from 6.25 to 50 μM; when combined with the other test agent, the MICs either increased or decreased. The MICs of MUC7 12-mer increased 4 fold when combined with Ca^2+^. After analysis of the data with FIC index model, FICi value of 4.5 was obtained, indicating antagonisms between MUC7 12-mer and Ca^2+^. In contrast, the MICs of MUC7 12-mer decreased 8 fold when combined with EDTA; FICi of 0.22 was obtained in this case, indicating a synergism between MUC7 12-mer and EDTA. In addition, Na^+^, Mg^2+^, and Zn^2+ ^decreased the MIC of MUC7 12-mer by 2 fold, while K^+ ^increased the MIC by 2 fold. However, instead of antagonism, an additivity or indifference (FICi 0.55 to 2.5) was observed in the combination of MUC7 12-mer and each K^+^, Na^+^, Mg^2+^, or Zn^2+^.

**Table 1:** Interaction of MUC7 12-mer in the combination with salts or EDTA against *C. albicans*

	MIC (μM)^a^			
			
Combination pairs	alone	combined	FIC^b^	FICi^c^
MUC7 12-mer	50	25	0.5	
Na^+^	>250	31	0.062	0.562 (ADD)^d^
				
MUC7 12-mer	50	100	2	
K^+^	>250	250	0.5	2.5 (IND)
				
MUC7 12-mer	12.50	50.00	4.00	
Ca^2+^	>160	80.00	0.50	4.50 (ANT)
				
MUC7 12-mer	12.50	25.00	1.00	
Mg^2+^	>160	80.00	0.25	1.25 (IND)
				
MUC7 12-mer	6.25	3.13	0.50	
Zn^2+^	160.00	7.50	0.05	0.55 (ADD)
				
MUC7 12-mer	18.75	2.35	0.13	
EDTA	75.00	6.25	0.09	0.22 (SYN)

^a ^Minimal inhibitory concentrations (MIC) was determined at the endpoint of MIC-0, which is defined as the lowest agent concentration showing no more than 10% growth in comparison to that of the growth control. The data represents the median obtained from three independent experiments.

^b ^FIC is a fraction inhibitory concentration that was calculated by dividing the MIC value of agent in combination by the MIC value of the agent alone.

^c ^FICi is a FIC index, indicating the sum of the FICs of the two agents in the pairs.

^d ^Interactions: SYN, synergism (FICi <0.5); ADD, additivity (FICi 0.5–1.0); IND, indifference (FICi 1.0 – 4.0); ANT, antagonism (FICi >4).

Interestingly, for growth inhibition of *S. mutans *and *E. coli *(data not shown), synergism (FICi 0.38) between MUC7 12-mer and EDTA, or additivity (0.56) between MUC7 12-mer and Zn^2+ ^was also observed. Instead of antagonism, the combination of MUC7 12-mer and Ca^2+ ^exhibited indifference (FICi 2.5) for the inhibition of *S. mutans *growth.

The physiological concentration of Ca^2+ ^in human whole saliva is 1–2 mM [10]. Ca^+2 ^may be the main inhibitory factor on the activity of MUC7 12-mer *in vivo*. In fact, as shown in Table 2, MIC of MUC7 12-mer against *C. albicans *increased from 18 to 200 μM as the concentration of Ca^2+ ^increased. Therefore, the effect of EDTA on the anticandidal activity of MUC7 12-mer in the presence of Ca^2+ ^was also determined. As pointed out above, the combination of MUC7 12-mer and EDTA exhibited a synergistic effect on the anticandidal activity. In the presence of Ca^+2 ^and EDTA (Table 2), MUC7 12-mer still showed strong activity, with the MIC decrease from 4 to 16 fold compared to the MICs in the presence of Ca^+2 ^without the EDTA. Synergism was still observed with FICi 0.22 to 0.31.

**Table 2:** Interaction of MUC7 12-mer in the combination with EDTA against *C. albicans *in the presence of Ca ^2+^

		MIC (μM)			
				
Ca ^2+ ^(mM)	Pair	alone	combined	FIC	FICi
0	MUC7 12-mer	18.75	2.35	0.13	
	EDTA	75.00	6.25	0.09	0.22
					
0.01	MUC7 12-mer	25	6.25	0.25	
	EDTA	50	3.13	0.0626	0.3126
					
1	MUC7 12-mer	100	6.25	0.0625	
	EDTA	100	25	0.25	0.3125
					
2	MUC7 12-mer	200	6.25	0.03125	
	EDTA	200	50	0.25	0.28125

All parameters (MIC, FIC, FICi) are defined in table 1 footnote.

### Killing activity of MUC7 12-mer peptide in the presence of sodium and potassium

**Figure 1:**
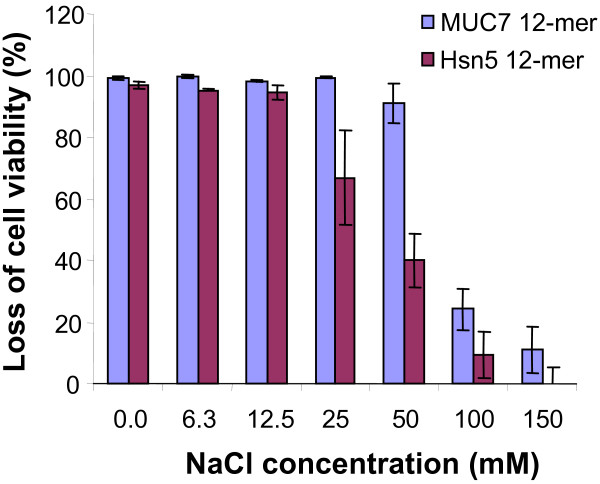
**Effect of sodium chloride on candidacidal activity of MUC7 12-mer and Hsn5 12-mer, determined by killing assay**. MUC7 12-mer (blue column) or Hsn5 12-mer (red column) (at a final concentration of 25 μM) were incubated with *C. albicans *(10^5 ^cells/ml) in 10 mM Na-PB containing NaCl (6.25–150 mM). The killing activities were determined by viable counting on SDA, comparing the number of colonies at each salt concentration to those of the agent-free control. Data represents two independent experiments, each performed in duplicate.

The effect of peptides on cell viability of *C. albicans *in the presence of NaCl is shown in Figure 1. MUC7 12-mer exhibits 100% killing activity in the presence of NaCl up to 25 mM, a physiological concentration of Na^+ ^in human saliva. Some decrease in the activity of the MUC7 12-mer was observed in the presence of 50 mM NaCl or higher. However, MUC7 12-mer still exhibited more resistance to NaCl than Hsn5 12-mer. In the presence of 50 mM NaCl, MUC7 retained 90% killing activity, while Hsn5 12-mer 40%. Additionally, MUC7 12-mer peptide was effective against *E. coli *in the presence of NaCl up to 150 mM (data not shown), a physiological concentration of Na^+ ^in plasma.

**Figure 2:**
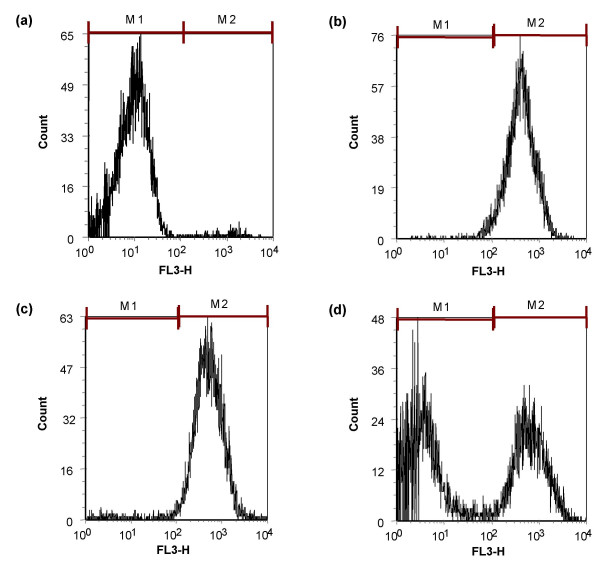
**Antimicrobial activity of MUC7 12-mer against *C. albicans *in the presence of potassium, monitored by flow cytometry**. Fluorescence distribution of the control (untreated) *C. albicans *cells (a); cells incubated for 90 min at 37°C with 25 μM MUC7 12-mer (b-d), in the absence of KCl (b), in the presence of 40 mM KCl (c), in the presence of 80 mM KCl (d). The cells were stained with propidium iodide (3 μg/mL). Susceptibility to MUC7 12-mer is shown as percentage of *C. albicans *with increasing fluorescence; 10,000 events were analyzed.

**Figure 3:**
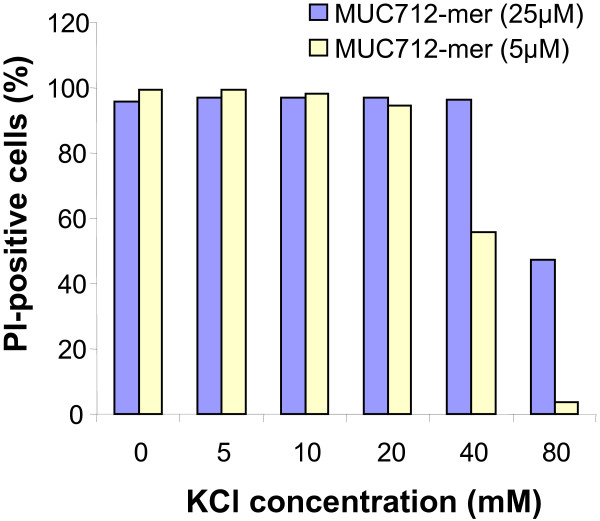
**Effect of potassium on anticandidal activity of MUC7 12-mer**. *C. abicans *DIS (10^6 ^cells/ml) was treated with 5 and 25 μM MUC7 12-mer in Na-PB containing KCl (5 – 80 mM) and PI fluorescence was analyzed by flow cytometry as described in Materials and Methods.

Figures 2 and 3 show the effect of K^+ ^on the anticandidal activity of MUC7 12-mer, which was determined with flow cytometry. Untreated cells stained with PI showed a very low intensity of fluorescence, expressed as negative (viable cells, Figure 2a) for PI. In contrast, MUC7 12-mer or ethanol (data not show) treated cells showed a high red fluorescence, expressed as positive (dead cells, Figure 2b) for PI. *Candida *cells treated with MUC7 12-mer in the presence of 40 mM KCl still showed PI fluorescence (Figure 2c) with 96% PI positive cells (Figure 3), although the PI positive cells deceased to 47% in the presence of 80 mM KCl (Figure 2d and Figure 3). Similarly, when treated with a low concentration of MUC7 12-mer (5 μM), more than 96% of *Candida *cells were killed in the presence of KCl at concentrations up to 20 mM, and 56% of cells were killed at 40 mM KCl.

### Effect of pH

**Figure 4:**
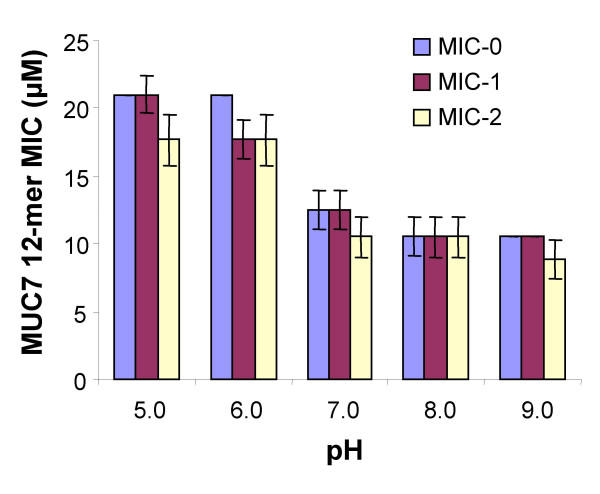
**Effect of pH on MIC of MUC7 12-mer against *C. albicans***. MIC-0, MIC-1 and MIC-2 indicate the lowest agent concentration showing no more than 10%, 25% and 50% growth respectively, in comparison to that of the agent-free control. The bars indicate the geometric means of two independent experiments, each performed in duplicate. The error bars show the 95% confidence intervals for the means.

The effect of pH on the activity of the peptide was established by determining the MIC of MUC7 12-mer against *C. albicans *DIS at pH values varying from 5 to 9 (Figure 4). There are no significant differences among the MIC-0, MIC-1, and MIC-2 (95% confidence intervals overlap). However, MICs of MUC7 12-mer were higher in acidic conditions than in neutral and basic conditions, and its antimicrobial activity was optimum at pH 8 (95% confidence intervals do not overlap).

### Effect of temperature

**Figure 5:**
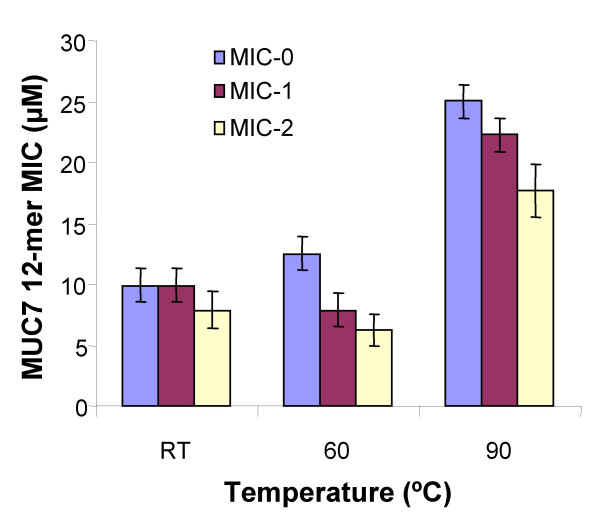
**The effect of temperature on MIC of MUC7 12-mer against *C. albicans***. MUC7 12-mer was heated for 30 min prior to determining the MIC values. RT: Room temperature. The bars indicate the geometric means of two independent experiments, each performed in duplicate. The error bars represent the 95% confidence intervals for the means.

The effect of temperature on the activity of MUC7 12-mer was determined by heating MUC7 12-mer for 30 min prior to determining the MIC values (Figure 5). MIC values of MUC7 12-mer did not significantly change after the peptide was pre-treated at 60°C; when pre-heated at 90°C, the peptide was still active although its MIC values slightly increased (MIC-0 = 25 μM, one dilution level). Temperature up to 60°C does not affect the activity of the peptide.

## Discussion

To have a therapeutic use against oral or systemic infections, antimicrobial peptides need to retain their activity in physiological conditions. Some antimicrobial peptides have a broad activity against fungi, bacteria and viruses. However, a major obstacle in their development as novel antibiotics is the antagonism between the peptides and ionic strength in their environment. As a result, the practical therapeutic use of antibiotic peptides is significantly impaired or attenuated. Many cationic antimicrobial peptides including β-defensins and the α-defensin HD-5 [11-16], lactoferricin B [17], histain 5 [18-20], human cathelicidin LL-37 [21], protegrins [22], and pleurocidin [23] are salt sensitive and reduce or lose their antimicrobial activity at elevated concentrations of mono- or divalent cations. The persistence of *P. aeruginosa *infections in the lungs of patients with cystic fibrosis is attributed to the susceptibility of epithelial antimicrobial peptides to salt antagonism [14]. The antibacterial effectiveness of lactoferricin B was reduced in the presence of Na^+^, K^+^, Mg^2+ ^or Ca^2+ ^ions, or in the presence of various buffer salts [17]. Hypertonic salt concentrations and heat-inactivated serum were found to be inhibitory to the bactericidal activity of Protegrin (PG-1), a broad spectrum antibiotic peptide isolated from porcine leukocytes [22]. Human beta-defensin-2 and cathelicidin LL-37 inhibit the growth of *P. aeruginosa in vitro*, but this activity is markedly reduced in the presence of tears [24]. In saliva, Histatin 5, a human basic salivary peptide with strong fungicidal *properties in vitro*, is salt sensitive and exerts low activity at high salt conditions [25]. The activity resumed after removing the salts by dialysis [19].

Saliva is vitally important for the normal functioning of healthy human beings. Saliva is hypotonic in relation to serum; the average concentration of Na in saliva is 6–26 mM, versus 140 mM in plasma. Ca^2+ ^and K^+ ^are the major salivary complex-forming ions [26]. The normal values of major cations and pH in mixed human saliva and plasma is summarized in Table 3. Since NaCl is the most predominant salt *in vivo*, the ability to resist this salt is significant for antimicrobial peptides to function under physiological conditions. Oral candidiasis is a superficial mucosal infection caused by *C. albicans *that is frequently associated with chemotherapy, organ transplantation, immunodeficiency virus infections, underlying disease states or medications that reduce salivary flow. A NaCl concentration of 6–26 mM has been reported to be present in the environment of the epithelial cells of the oral cavity (Table 3). In this study, the antagonism in terms of growth inhibition between Na^+ ^and MUC7 12-mer was not observed by the checkerboard test (Table 1). More importantly, MUC7 12-mer still exhibited anticandidal activity at the human salivary Na^+ ^concentration (less than 26 mM; Figure 1). This suggests that MUC7 12-mer peptide is active for oral candidiasis in oral physiological conditions. This is consistent with the observation of our previous study using mouse model of oral candidiasis *in vivo *[27]. The other reported NaCl-resistant peptides include clavanins (histidine-rich, amidated alpha-helical antimicrobial peptides) [28], CP26, CP29, CEME, CEMA (the analogues based on the insect cecropin-bee melittin hybrid peptide) [29], P18 (KWKLFKKIPKFLHLAKKF, an alpha-helical antimicrobial peptide) [30], cathelicidin CAP18, SMAP29 [31], and modified forms of LL-37, such as pentamide-37 [32]. Ponti *et al.* reported that the cyclic and linear analogues of esculentin-1, an antimicrobial peptide from amphibian skin, have a strong killing activity against gram-negative bacteria, and the activity is not affected by salts [33].

**Table 3:** The normal values of cations and pH in mixed human saliva and plasma

	Saliva (mM)	Plasma (mM)
Ca^2+^	1–2	2.5
Mg^2+^	0.2–0.5	0.5–1.0
Na^+^	6–26	140
K^+^	14–32	4
NH_4 _^+^	1–7	0.03
pH	6.7	
pH range	6.2–7.6	

Reference: [10]

Nuding, *et al*. reported that the antibacterial effect of the isolated cationic extracts from human intestinal biopsies was diminished towards *E. coli *with 150 mM NaCl [34]. Our study (data not shown) demonstrated that MUC7 12-mer exerted strong activity towards *E. coli *HB101 up to 150 mM NaCl. This suggests a potential for MUC7 12-mer as a chemotherapeutic agent for the systematic infection with *E. coli *without being affected at physiological salt concentrations.

KCl is one of the salts affecting the antimicrobial activity. However, to our knowledge, the reported information regarding the effect of KCl on the antimicrobial peptide activity is limited. KC is not a principal salt in plasma, but is predominant in saliva (14–32 mM) [26]. Thus, the role of KCl should not be neglected for the anticandidial activity of peptides. Previously, it has been observed that extended indolicidins, ^β^-sheet gramicidins, and looped and linear bactenecins are all quite sensitive to KCl [35]. In this study, the antagonistic effects of KCl was not observed on MUC7 12-mer, which still showed high anticandidal activity in the presence of 40 mM KCl, the concentration higher than its physiological concentration in saliva.

Ca^2+ ^is a major divalent cation in saliva, a potent inhibitor of histatin 5 candidacidal activity at physiological concentrations, and may be the primary ion responsible for the masking effect of saliva [36]. Our previous study showed that Ca^2+ ^exhibited inhibitory effect on the fungicidal activity of MUC7 20-mer (a peptide extended by 8 aa residues at the N-terminus of the MUC7 12-mer) [3]. In this study, we found that Ca^2+ ^is the only ion that exhibited an antagonistic effect on MUC7 12-mer against *C. albicans*.

It has been reported that Mg^2+ ^also inhibits the activity of antimicrobial peptides, but the inhibition potency is lower that that of Ca^2+^. For example, Mg^2+ ^at the physiological concentration (less than 1 mM) does not change the antibacterial activity of the Sphe-2, a β-defensin from the king penguin stomach, against *S. aureus*, and the growth of *E. coli *was altered by 2 fold in the presence of 1 mM MgCl_2_[37]. We have demonstrated (in the previous study) that more than 5 mM MgCl_2 _exhibited inhibitory effect on the candidacidal activity of MUC7 20-mer. The activity, however, was not altered in the presence of 1 mM MgCl_2 _[3]. In this study, we demonstrated that Mg^2+^, unlike Ca^2+^, did not show an antagonistic effect on MUC7 12-mer against *C. albicans*.

Zn^2+ ^does not exist in saliva, but Zn^2+ ^can enhance the antimicrobial activity of peptides. It has been reported that poly(arginyl-histidine) peptide, which shows activity against a broad range of bacteria and fungi, lost its activity under conditions of high ionic strength. Zn^2+ ^can specifically change the circular dichroism spectra of this peptide and restore its antimicrobial activity under high ionic strength conditions [38]. Melino *et al*. reported that Zn^2+ ^ions selectively induce salivary histatin-5 to fuse negatively charged vesicles through a zinc-binding motif present in its functional domain, and stated that the action of this antimicrobial peptide is likely mediated by the presence of zinc ions [39]. Our results are consistent with the above studies in that Zn^2+ ^exhibits an additive effect on the antimicrobial activity of MUC7 12-mer. However, the mechanism of this effect in not known.

EDTA (ethylenediamine tetraacetic acid), which chelates divalent cations, is approved by the FDA as a preservative in packaged foods, vitamins, and baby food. EDTA has been widely used in many areas. In medicine, EDTA is used in chelation therapy for acute hypercalcemia and for treating mercury or lead poisoning. EDTA is also used as an anticoagulant for blood samples, and as an anticollagenase to prevent the worsening of corneal ulcers in animals. In dentistry, EDTA is used as a root canal irrigant to remove organic and inorganic debris (smear layer) [40,41]. Sen *et al*. demonstrated that EDTA exhibited the highest antifungal activity against *C. albicans*, compared with those of routine antifungal drugs [42]. Our previous study showed that that EDTA enhanced the antifungal activity of MUC7 peptides in human saliva mainly by chelating the divalent cations (such as Ca^+2^) [8]. In support of this discovery, the results of this study indicated that EDTA has a synergistic effect on the antimicrobial activity of MUC7 12-mer (Table 1), and abrogates the activity that was inhibited by Ca^2+ ^(Table 2). These findings suggest that concurrent therapies with EDTA and MUC7 12-mer may be of value in patients with oral fungal or bacterial infections, although further studies *in vivo *are needed to examine the safety, tolerance, and optimal dosing of EDTA in the treatment of these infections. The mechanism for the EDTA action is likely that it competes with microorganism for any of the trace iron and Ca^2+ ^ions that are essential to the maintenance of their life cycle [43] or it disturbs the integrity of bacterial outer membrane [44]. It may be also due to the fact that EDTA prevents the peptide degradation by the metalloprotease [45].

In addition to salt sensitivity, antimicrobial peptides are also pH-dependent. Lee *et al*. [28] demonstrated that the activities of histidine-rich, amidated alpha-helical antimicrobial peptides were substantially greater at pH 5.5 than at pH 7.4. In another study, Minahk *et al*. also showed that the antilisterial activity of enterocin CRL35 was higher at acidic than neutral or basic conditions [46]. In contrast, our study showed that the antimicrobial power of MUC7 12-mer peptide was enhanced in neutral or slightly alkali media, although the peptide acted in a broad range of pH. Therefore, saliva with pH range 6.2–7.6 appears to be the suitable environment for the action of MUC7 12-mer. Similar observations have been reported for other antimicrobial peptides [47,48].

Temperature is also one of the important factors for the therapeutic application of the peptide. Some antimicrobial peptides retained their activity when pre-heated [47]. MUC7 12-mer is also thermo-stable. It retained significant antimicrobial activity after incubation at 60°C for 30 min. This thermal stability is probably due to this peptide's very simple secondary structure that cannot be denatured.

## Conclusion

At oral physiological concentrations, the antimicrobial activity of MUC7 12-mer is not affected by Na^+^, K^+^, or Mg^2+^, but is inhibited by Ca^+2^, which has an antagonistic effect on MUC7 12-mer. However, this inhibition can be reversed in the presence of EDTA, as EDTA exhibited a synergistic effect when combined with MUC7 12-mer. This peptide exerts optimum anticandidal activity at neutral or slightly basic conditions. MUC7 12-mer peptide is stable, and temperature up to 60°C has not affected its activity. These findings suggest that MUC7 12-mer peptide, especially in combination with EDTA, could be potentially applied as an alternative therapeutic agent for the treatment of human oral candidiasis.

## Competing interests

The author(s) declare that they have no competing interests.

## Authors' contributions

GXW designed the study, performed experimental work, collected and analyzed the data, and drafted the manuscript. ANC was involved in performing killing assay and critical reading of the manuscript. LAB conceived the study, provided guidance and supervision throughout the study and participated in writing of the manuscript. All authors read and approved the final manuscript.
